# Effects of Activation Barriers on Quenching to Stabilize Prebiotic Chemical Systems

**DOI:** 10.3390/life14010116

**Published:** 2024-01-12

**Authors:** Qianyi Sheng, Ben Fredrick Intoy, J. W. Halley

**Affiliations:** School of Physics and Astronomy, University of Minnesota-Twin Cities, Minneapolis, MN 55455, USAintoybf@vt.edu (B.F.I.)

**Keywords:** astrobiology, prebiotic chemistry, origin of life, hydrothermal systems, quenching

## Abstract

We have previously shown in model studies that rapid quenches of systems of monomers interacting to form polymer chains can fix nonequilibrium chemistries with some lifelike properties. We suggested that such quenching processes might have occurred at very high rates on early Earth, giving an efficient mechanism for natural sorting through enormous numbers of nonequilibrium chemistries from which the most lifelike ones could be naturally selected. However, the model used for these studies did not take account of activation barriers to polymer scission (peptide bond hydrolysis in the case of proteins). Such barriers are known to exist and are expected to enhance the quenching effect. Here, we introduce a modified model which takes activation barriers into account and we compare the results to data from experiments on quenched systems of amino acids. We find that the model results turn out to be sensitive to the width of the distribution of barrier heights but quite insensitive to its average value. The results of the new model are in significantly better agreement with the experiments than those found using our previous model. The new parametrization of the model only requires one new parameter and the parametrization is more physical than the previous one, providing a chemical interpretation of the parameter *p* in our previous models. Within the model, a characteristic temperature $T_{c}$ emerges such that if the temperature of the hot stage is above $T_{c}$ and the temperature of the cold stage is below it, then the ‘freezing out’, in a quench, of a disequilibrium ensemble of long polymers is expected. We discuss the possible relevance of this to models of the origin of life in emissions from deep ocean rifts.

## 1. Introduction

The likelihood of natural formation of an initial genome in ‘genome first’ models of prebiotic evolution appears to be nearly impossible ‘Eigen’s paradox’ [1]). This has motivated interest in alternative models in which the early phases of prebiotic systems are characterized by collections of polymers exhibiting lifelike behavior and storing information collectively without a central genome. Estimates of the likelihood of the random natural formation of such entities, of which prions and amyloids [2,3,4,5,6] are often mentioned as examples, are probably higher, but how likely they are to form prebiotically is poorly understood and a major issue in evaluating such models.

In previous work [7], we showed that a coarse-grained model of putative polymer prebiotic chemistry suggested quenching of a collection of such interacting monomers from a high temperature to ambient conditions as a prebiotic process. Such quenches could allow for a wide exploration of the space of polymer combinations in a high-temperature environment before the following quench fixed a nonequilibrium state which could have some metastable lifelike properties. We cited experimental work [8], refs. [9,10,11], which showed that a form of quenching (different in the two sets of experiments) did indeed enhance polypeptide formation in solutions of amino acids. We suggest that such quenching might occur in ocean trenches, similar to the hypotheses of others [12,13,14] that prebiotic chemistry might have occurred in tectonic faults. Other possible sites of repeated quenching in early Earth include hot springs, beaches and lagoons in proximity to volcanic activity, hydrothermal sediments, shallow water hydrothermal vents and heated rock pores [15,16,17,18,19].

However, our quenching model had some inadequacies: all reactions (ligation and scission) were assumed to be barrierless and the model was characterized by a parameter *p*. *p* was defined to be the probability of the presence of any possible reaction in the chemical network as originally introduced in similar models by Kauffman [20,21]. In the calculations reported by Kauffman and coworkers [20,21], an ensemble of artificial chemical networks was constructed in which any possible reaction occurred with probability *p*. However, the relationship of *p* with the chemical and physical processes occurring in real systems was somewhat unclear.

Here, we report simulation results from a modified model which addresses these inadequacies: we have eliminated *p* and replaced it with two parameters characterizing the distribution of barrier heights for polymer scission reactions. The statistical distribution of barrier heights is introduced and sampled to parametrize the temperature dependence of the reaction rates. The distribution used is Gaussian, consistent with the limited information available experimentally concerning barrier heights for peptide bond hydrolysis as measured in nonbiological contexts [22,23]. Parameters from nonbiological systems are selected because rates in modern biological systems are determined by highly evolved processes involving enzymes which cannot be assumed to exist in prebiotic environments. Nevertheless, we stress that the data on peptide bond scission via hydrolysis which we use are very limited and the parameters could be different in an early Earth environment. A Gaussian distribution would be expected if the effective rates of scission were a product of the rates, each of the Arrhenius form, of a large number of steps, each randomly distributed. However, given the limited information available, the Gaussian distribution assumed here must be regarded as a hypothesis of the model. We have not fully explored the consequences of other assumptions concerning this distribution. Our qualitative results depend mainly on the fact that the distribution of the effective rate *v* is zero at exactly v=0 and has a sharp maximum near v=0 as discussed below. The mean and standard deviation of the distribution are initially selected in a way that is consistent with what is known experimentally but are then varied to fit the data from quenching experiments reported in references [8,9,10,11].

We show that the model produces a distribution of reaction rates which is very similar to the one implied in models parametrized by *p*, though the distribution is temperature (and pH) dependent. A temperature $T_{c}$ emerges such that if the temperature of the hot environment before the quench is above $T_{c}$ and the temperature of the cold environment is below $T_{c}$, then the quench leads to a disequilibrium ensemble of long polymers. $T_{c}$ is estimated from data on the barriers of peptide bond hydrolysis to be around the boiling point of water, but it depends logarithmically on the time which the system spends in the hot part of the quench. A comparison with experiments in [8,9,10,11] shows that the data are in significantly better agreement with the new model than they were using the previous one [7]. The resulting final states after quenching are farther from equilibrium than in those previous calculations [7].

All the parameters are accessible in principle from experiment. The least well known of them in most cases are the time which the system spends in the high-temperature environment before the quench and the width of the Gaussian distribution of barrier heights. We use a fitting procedure to establish bounds on the possible values of these parameters which are most consistent with the model and the experiments. The theoretical results are quite sensitive to the width of the activation barrier distribution but are much less sensitive to its (better known) mean. Oscillations in the number of polymers of length *L* as a function of *L* observed after quenching in the experiments of references [8] and, to a lesser extent, refs. [9,10,11], are reproduced in the model simulations and an analysis is presented to provide insights into this phenomenon.

We also discuss the application of the model to quenches which occur in ocean trenches, for example, in smokers. The parameters are significantly different to those in the laboratory experiments; the ‘dwell times’ that the fluid spends in a high-temperature environment before quenching are significantly longer (up to years) in most of these cases and the temperatures are often much higher (up to 600K in some cases). We show that the model predicts formation of mainly long polymers in the case of polypeptides under suitable combinations of such conditions. We find some support for this prediction in one oceanographic report [24] but it could be tested much more extensively via observational data. We discuss the possible implications for origin of life models which postulate origins in ocean rifts.

The next section briefly describes our previous model [7] and how it was modified to take account of activation energies. In Section 3, we provide some simulations and analytical comparisons of the new model with our previous one. The Section 4 describes a detailed analysis of the experiments using these tools and Section 5 provides a description of the application to quenches in ocean rifts.

In the conclusions, we discuss the implications for a scenario in which lifelike assemblages of proteins or other biopolymers might have formed on very rare occasions and been naturally selected from millions of quenches of aqueous solutions emerging from ocean trenches or ridges on early Earth and we suggest directions for future work.

## 2. Model and Simulation Methods

The model used for quench simulations including activation energies is the same as that used to obtain the results reported in [7], except that a different distribution of reaction rates is used arising from the distribution of barrier heights for polymer scission as described qualitatively above and in more detail below. As in [7,25,26] and elsewhere [20,21], artificial chemistries associated with abstracted polymers are generated, consisting of strings of digits representing monomers. The polymers undergo scission and ligation. However, unlike previous work, the present model does not have a parameter *p* which controls the probability that, in a given realization, any possible reaction involving polymers up to a maximum length of $l_{max}$ is included in the chemical network. Instead, we introduce a distribution of reaction rates, determined by a Gaussian distribution of activation energies as described below. This permits us to define an effective $p_{eff}$, which is a function of the temperature, the time the system spends in contact with a reservoir at that temperature, and the pH.

We then use $p_{eff}$ as *p* was used in [7] to construct an ensemble of artificial chemical networks and study their dynamic behavior as covalent bonds form and break due to scission and ligation. Each reaction in the network is randomly assigned one enzyme from the species present in the network, as in [7]. The algorithm used in the simulations reported here is nearly the same as that used in our previous work, but is different in some details and is summarized in Appendix A. An important difference is that we do not eliminate networks that do not contain reaction paths from the food set to at least one polymer of length $l_{max}$ (which we previously called ‘unviable’) when performing dynamical simulations in the present work. This is because we wish to simulate both natural conditions and experiments in which such elimination does not take place. We assume here that the system is ‘well mixed’ and no effects of spatial diffusion are considered.

As in [7,26], we assign to any ‘polymer’ (string) of length *l* an energy −(l−1)Δ, where Δ is a real number which is the bonding energy between two monomers. These ‘bonding energies’ determine the thermodynamic driving force for bond formation and are negative in the application to peptide bonds. They are assumed to be the same for pairs of all types of monomers and are to be distinguished from the activation energies for bond breaking discussed below. Monomers are assigned ‘types’ of which there are *b*, an integer. For proteins, b=20, for nucleic acids, b=4, and in our simulations, b=1,…,8. The total number of possible polymeric species (distinct series of ’types’) of length *L* is then $b^{L}$. The total energy *E* of any population $\left\{n_{m}\right\}$ of polymers, in which $n_{m}$ is the number of polymers of species *m*, is $E=-\sum_{L=1}^{l_{max}}\left(L-1\right)N_{L}\Delta$. Here, $N_{L}=\sum_{m\;of\;length\;L}n_{m}$ is the same set of macrovariables used in [7,25,27]. The total number of polymers *N* is $N=\sum_{L=1}^{l_{max}}N_{L}$. The parameter β is defined as $\beta \equiv 1/k_{B}T$, where *T* is the absolute temperature, which we assume to be positive so that, as we take Δ<0, the relevant parameter βΔ<0.

To address the central problem of the prebiotic origin of life as enunciated by Eigen [1] and many others, we focus in our models on the configurational entropy associated with a coarse-grained description of the state of a system of polymers in which the numbers of molecules of each length *L* between L=1 and $L=l_{max}$ is $N_{L}$. This entropy is [27,28]

(1) $$\begin{array}{c} S/k_{B}=\sum_{L}\left[\ln\left(\left(b^{L}-1+N_{L}\right)!\right)-\ln\left(N_{L}!\right)-\ln\left(\left(b^{L}-1\right)!\right)\right] \end{array}$$

The −1 in the expression $\ln(\left(b^{L}-1+N_{L}\right)!)$ arises from the counting statistics which, coincidentally, turn out to be the same as those for Bose fluids and are described, for example, in references [27,28]. When b=1, this configurational entropy is zero because there is only one configurational state. To model experimental systems, we include another factor in the term $b^{L}$, as described later in this section, and the entropy becomes non zero when b=1. However, in the present paper, we only consider simulations and experimental systems for which b>1.

In our simulations, the polymers of interest are not in equilibrium; however, in addition to the nonequilibrium distributions calculated from kinetics, we also calculate the equilibrium distributions $\left\{\bar{N_{L}}\right\}$ associated with local equilibrium as well as the values $\left\{\bar{N_{L}}\right\}$ associated with the system in equilibrium with a temperature bath at temperature *T*. The two sets of equilibrium values $\left\{\bar{N_{L}}\right\}$ are recalculated continuously during the simulations. For b>1, these distributions are both of the form:

(2) $$\bar{N_{L}}=\frac{b^{L}-1}{\exp(-\beta \mu -\beta \Delta (L-1))-1}$$

To determine the isolated (equivalently local) equilibrium state, we compute β and μ from the known energy *E* and polymer number *N* by solving (on the fly during simulations) the equations

(3) $$E=-\sum_{L=1}^{l_{max}}\left(L-1\right)\bar{N_{L}}\Delta$$

and

(4) $$N=\sum_{L=1}^{l_{max}}\bar{N_{L}}$$

together with (2). On the other hand, for an equilibrium with an external thermal bath, we fix β and compute μ from the solution of Equations (2) and (4).

For comparison with laboratory experiments and oceanographic measurements on polypeptide formation in quenches, as described in Section 4 and Section 5, we take entropic account of the dilution of the polymers in the experimental samples, as we did in the work reported in [29]. A reformulation is convenient because the experiments and observations report molecular concentrations, not absolute numbers of molecules. We introduce a microscopic length $R_{0}L^{\nu }$, where $R_{0}$ is a length related to the polymer persistence length and ν is an index which would be 1/2 for a random walk. The entropy $S/k_{B}$ becomes

(5) $$S/k_{B}=\sum_{L}\left[\ln\left(\left(N_{L}+G_{L}-1\right)!\right)-\ln(\left(N_{L}!\right)-\ln\left(\left(G_{L}-1\right)!\right)\right]$$

The term $G_{L}=b^{L}V/v_{p}L^{3\nu }$ is taking account of the number of configurational ways in which a polymer of length *L* can be formed if *b* types of monomer are available (the factor $b^{L}$) and also of the approximate number of ways that such a polymer may be found in a sample of volume *V* (the factor $V/v_{p}L^{3\nu }$). In the latter factor, $v_{p}=R_{0}^{3}$ and the values of $R_{0}$ and ν were taken from reports of light scattering experiments on denatured proteins to be $R_{0}=1.927$ and ν=0.588 [30]. The model summarized by (5) is physically equivalent to the one described in (1) except for the factor $V/v_{p}L^{3\nu }$ in $G_{L}$.

Maximizing the entropy, as described in [29], we find

(6) $$\bar{N_{L}\left(v_{p}/V\right)}=\frac{b^{L}/L^{3\nu }-\left(v_{p}/V\right)}{\exp(-\beta (e,\rho )\mu (e,\rho )-\beta (e,\rho )\Delta (L-1))-1}$$

and

(7) $$ev_{p}=-\sum_{L=1}^{l_{max}}\left(L-1\right)\bar{N_{L}\left(v_{p}/V\right)}\Delta$$

and

(8) $$\rho v_{p}=\sum_{L=1}^{l_{max}}\bar{N_{L}\left(v_{p}/V\right)}$$

which are expressed in terms of the experimentally accessible quantities ρ, the volumetric polymer density, and *e*, the volumetric energy density. To determine the equilibrium state resulting from equilibrium with a temperature bath, we fix β and determine μ by solution, on the fly, using Equation (8) together with (6).

After a network is formed according to the procedure reviewed in Appendix A, it is regarded as fixed. Barrier heights are selected and fixed for each reaction in it and a set of small molecules (we use dimers and monomers) is populated as an initial ‘food set’. Then, the formation (ligation) and scission of longer polymers follow dynamically in a separate dynamical simulation guided by the Master Equation [7,20,21,25,26].

(9) $$\begin{array}{cc} dn_{l}/dt= & \sum_{l^{\prime },m,e}[v_{l,l^{\prime },m,e}\left(-k_{d}n_{l}n_{l^{\prime }}n_{e}+k_{d}^{-1}n_{m}n_{e}\right)\\ & +v_{m,l^{\prime },l,e}\left(+k_{d}n_{m}n_{l^{\prime }}n_{e}-k_{d}^{-1}n_{l}n_{e}\right)]. \end{array}$$

Here, $n_{l}$ is the number of polymers of species *l*, $v_{l,l^{\prime },m,e}$ is proportional to the rate of the reaction l+l′e→m, *e* denotes the enzyme, *l* and $l^{\prime }$ denote the polymer species combined during ligation or produced during cleavage, and *m* denotes the product of ligation or the reactant during cleavage.

The dynamical model in (9) assumes that ligation and scission occur in single chemical steps. This is a simplification, but at least in our application to peptide bond scission, the Arrhenius form found experimentally in [23] suggests that a rate-limiting step determines the temperature dependence of the total rate, consistent with the form we have chosen for the temperature dependence of the total rate described below.

In (9), we have assumed that the rate constant $1/k_{d}$ for ligation reactions is the reciprocal of the constant $k_{d}$ for scission. This is not, in general, expected to be true, but we only found information on the rate of scission in the applications of interest. With (9), we can determine $k_{d}$ on the fly during dynamical simulations by requiring that the terms in (9) will obey a detailed balance when the system is in equilibrium, as we have applied in previous models [7,26]. The detailed balance condition is

(10) $$k_{d}^{2}=\bar{n_{m}}/\left(\bar{n_{l}}\;\bar{n_{l^{\prime }}}\right)$$

where, in the simulations reported here, the equilibrium distributions $\left\{\bar{n_{l}}\right\}$ in the last expression are always taken to be those associated with equilibrium with an external thermal bath with a fixed parameter β. The factors $k_{d}$ and $1/k_{d}$ then assure that the model is driven toward equilibrium and will reach it if not impeded by kinetic blocking or by the regular supplementation of molecules in the initial ’food set’ of monomers. Note that the number of equations represented by (9) is $b^{l_{max}}$ and each is of third order in the polynomials on the right. No analytic solution is possible, except in special cases like the one considered in Appendix A, in which the number of monomers is assumed to be much larger than the number of longer polymers. (In the simulations reported here, $b^{l_{max}}$ is characteristically of order $10^{4}$.) We use the well-known Gillespie algorithm [31] to stochastically simulate the polymer population statistics implied by (9).

Activation energies, which are the new feature in the model described here, enter in the parameters $v_{l,l^{\prime },m,e}$ or $v_{m,l^{\prime },l,e}$, which are assigned from a probability distribution:

(11) $$\begin{array}{cc} dP(v)/dv= & \\ -\frac{{\left(2/\pi \right)}^{1/2}}{\sigma \beta vs.(1+erf\left(\bar{\Delta_{a}}\sqrt{2}/\sigma \right))} & exp[-{\left(\ln vs.+\bar{\Delta_{a}}\beta \right)}^{2}/2{\left(\sigma \beta \right)}^{2}] \end{array}$$

which follows from the assumptions that the (normalized) rates *v* are of the form $=e^{-\beta \Delta_{a}}$ and the activation energies $\Delta_{a}$ are distributed in a Gaussian distribution with mean $\bar{\Delta_{a}}$ and variance σ but restricted (see Appendix C) to $\Delta_{a}>0$. (Note that this is the probability distribution for the rates of reactions represented by the factors *v* in (9); it does not refer directly to the distribution of particle populations.) In the applications to polypeptides discussed in the following sections, we used data from [23], reporting experiments on hydrolysis of glycine–glycine bonds, to fix $\bar{\Delta_{a}}$.

**Figure 1:**
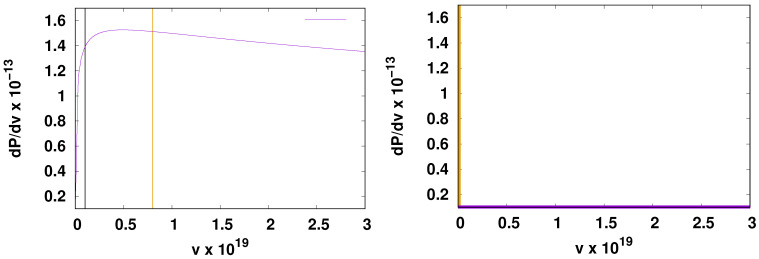
**Left panel**: An example of the probability distribution of rates *v* in the new model using $\Delta_{a}\beta =12,021,\sigma \beta =0.12\Delta \beta$. The temperature was taken to be 390 K. The vertical lines indicate two possible values of the parameter $1/(f_{a}t_{exp})$. We fix $f_{a}$ from [23]. In each case illustrated by the vertical lines, the area under the dP/dv curve between 0 and the line has a value of $1-p_{eff}$. The two vertical lines bracket the value $v_{max}$, where dP/dv has a maximum as discussed in the text. **Right panel**: Probability distribution in our previous model. The peak at v=0 in the previous model is actually a delta function at v=0 with integrated weight 1−p and the horizontal line just above the origin represents the constant probability density allowed for all values of *v* in the range of (0:1] (zero excluded). In both panels, only a small portion of the entire range of *v*, which extends to v=1 in both cases, is shown.

We show an example of the form of this distribution near v=0 in Figure 1, where it is compared with the distribution of factors *v* used in our previous models. (The latter was simply a delta function at v=0 with weight (1−p) plus a constant = p for 0<v≤1.) The following similarities and differences are noted: Similarly, there is a sharp spike in the probability distribution near *v* = 0 followed by a long tail. The range of *v* values is [0,1] as before, meaning that the rates are related to physical units in experiment by multiplying the rates by the prefactor in the Arrhenius expression for the activated rate. For later reference, we denote this prefactor by $f_{a}$. The differences include the fact that the sharp spike in our former models was exactly at v=0, whereas here the rate at exactly zero has zero weight and the position of the peak at low *v* is temperature-dependent, moving to higher values and broadening at higher temperatures.

In the case of our previous distribution, the peak at v=0 could be described as a delta function with weight 1−p, which is the probability that a reaction has zero rate and can be left out of consideration in forming networks. However, we cannot use this strategy with the present model, in which all reactions have rates with a finite weight, even though some of the rates are very small. The reason that these low-rate reactions can be neglected is that the experiment or natural evolutionary process will in any case occupy a finite time, and rates which are extremely small on that time scale can be neglected. (This consideration can be relevant in real systems; for example, the time for hydrolysis of some peptide bonds in pure water without enzymes has been estimated experimentally to be as much as one hundred years [23].)

However, in the simulation, if we do not take account of the actual time in the experiment or natural event, the simulation will simply cut off the slow reactions by default on a time scale set by the length of the run. Furthermore, by keeping all the reactions, the list of reactions would be very long, the reaction networks would be filled with many irrelevant reactions, and a lot of computation time would be spent rejecting these irrelevant reactions. In the following, we describe our procedure for taking these considerations into account in the calculations which follow. These procedures, and the introduction of the effective number $p_{eff}$ which arises from them, have the following advantages: (1) they provide a means to explicitly control the time which the system spends in contact with hot and then with cold reservoirs during quenching in the theory and simulations; (2) they allow for a physical interpretation of the parameter *p* in Kauffman-like chemical network models of prebiotic evolution; and (3) they permit simulations which only spend computational time on reactions which actually occur and the codes are therefore more efficient than alternative simulation methods would be.

To quantify these considerations and produce a simulation which is relatively efficient and takes them into account, we introduce a time $t_{exp}$ which characterizes the time during which the experiment or natural process being modeled is in the hot stage before the quench. Values of $t_{exp}$ will be discussed in more detail below, but we note here that they are quite well defined for laboratory experiments and are usually macroscopic (minutes to hours). For natural evolutionary processes, they are not known because we do not know exactly what these evolutionary processes are. However, if, for example, the idea that the essential processes occur as hot water exits ocean trenches or tectonic vents is relevant, then the relevant time for the high-temperature period before the quench would be the time that the solution remains hot. In oceanographic circulation models, this time is usually taken to be up to a few hundred degrees Celsius, because the water is under a high enough pressure not to boil at these temperatures. Temperatures of that order of magnitude have been observed in the postulated environments. The times can be estimated from measured flow rates and are reported [32] to be very heterogeneous, but are mainly in the range of 1 to 10^5^ yr.

Having chosen the parameter $t_{exp}$ from such considerations, we define an effective $p_{eff}$ by excluding reactions which do not have time to occur in the available time $t_{exp}$. This is achieved by requiring that all reactions for which $(t_{exp}\left(vf_{a}\right))>1$ be neglected. The factor $f_{a}$ converts *v* to physical time units and the requirement is that the reactions do not have time to occur in the available time. The cutoff value of 1 is somewhat arbitrary, but the cutoff is expected to be of order 1. To obtain a value for $p_{eff}$, we then integrate the probability distribution (11) for *v* from $1/(f_{a}t_{exp})$ to 1, as described in Appendix C. The resulting weight is set equal to $p_{eff}$. We illustrate some of these points in Figure 1.

**Figure 2:**
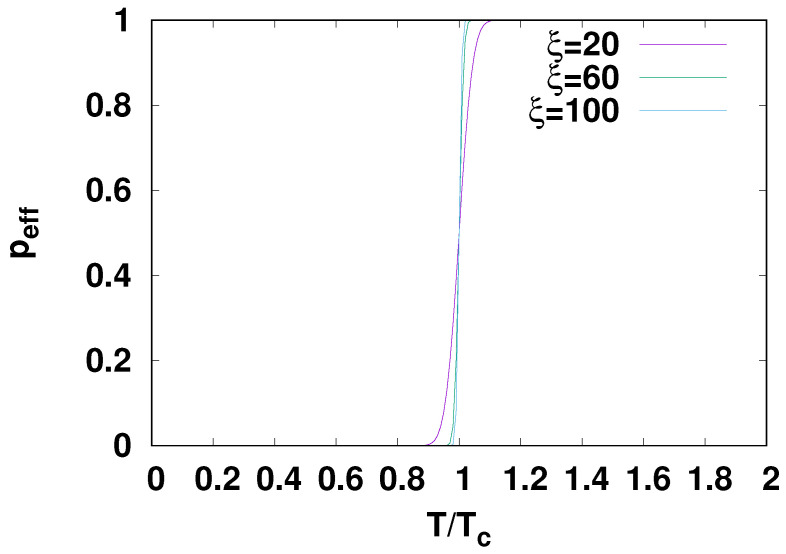
Dependence of $p_{eff}$ on the temperature for several values of the parameter ξ.

**Figure 3:**
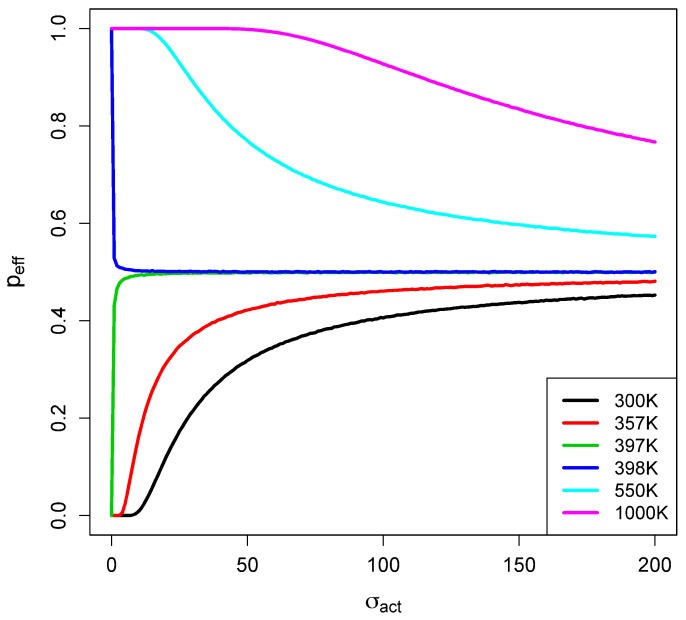
$p_{eff}$ versus σ at various values of the temperature as discussed in the text. Note that the sign of the derivative with respect to σ changes when the temperature passes through $T_{c}$.

The dependence of $p_{eff}$ on the temperature of the bath in which the simulations take place is shown in Figure 2, and its dependence on the width σ of the assumed Gaussian distribution of barrier heights is shown in Figure 3. Values of the parameters $f_{a}$ and $t_{exp}$ roughly consistent with the experiments considered later were used. An interesting feature is the sharp change in behavior at a particular temperature, which we denote $T_{c}$, at which the sign of the derivative of $p_{eff}$ with respect to σ changes. For temperatures below $T_{c}$, $p_{eff}$ decreases with decreasing σ and for a small enough σ, $p_{eff}$ becomes zero, meaning that the network has not had time for any reactions to take place. For temperatures above $T_{c}$, the values of $p_{eff}$ increase with decreasing σ and will saturate at 1 at a high enough temperature and low enough σ values. This behavior is quite easily understood, as seen in Figure 1, and unlike the Kauffman model distribution with which it is compared there, the distribution has a maximum as indicated in the top panel of the figure. The value of *v* at the maximum is easily computed from Equation (11), giving

(12) $$\ln v_{max}=-{\left(\sigma \beta \right)}^{2}-\bar{\Delta_{a}}\beta .$$

If this maximum value lies below the cutoff value $-\ln(f_{a}t_{exp})$, then when σ decreases, more of the probability weight will lie below the cutoff, $1-p_{eff}$ will grow, and $p_{eff}$ will shrink with decreasing σ. On the other hand, if the maximum lies above $-\ln(f_{a}t_{exp})$, then decreasing σ causes $p_{eff}$ to grow because increasingly less of the weight lies below the cutoff, causing $1-p_{eff}$ to shrink. The first case corresponds to low temperatures and the second to high temperatures. The critical temperature at which the behavior changes is approximately found by setting $\ln v_{max}$ in the preceding equation to $-\ln(f_{a}t_{exp})$ and solving for the critical temperature. A few details are supplied in Appendix C. When $\frac{\sigma^{2}\ln\left(t_{exp}f_{a}\right)}{\bar{\Delta_{a}^{2}}}$ is much less than 1, we find the physically relevant solution to be

(13) $$k_{B}T_{c}=\frac{{\bar{\Delta }}_{a}}{\ln(t_{exp}f_{a})}$$

This calculation illuminates the meaning of the temperature $T_{c}$ in the model. In the calculation in Appendix C of $p_{eff}$ as a function of the parameters $\bar{\Delta_{a}},\sigma ,t_{exp}$, and $f_{a}$, one finds that $T_{c}$ as defined by (13) again appears when $\frac{\sigma^{2}\ln\left(t_{exp}f_{a}\right)}{\bar{\Delta_{a}^{2}}}<<1$. Using this calculation as described in Appendix C, we find the following expression for $p_{eff}$ in terms of the error functions, with arguments depending only on $T_{c},T$, and $\xi =\sqrt{2}\left(\bar{\Delta_{a}}/\sigma \right)$.

(14) $$p_{eff}=\frac{erf\left(\xi \right)-erf(\xi \left(1-T/T_{c}\right))}{erf(\xi )+1}$$

**Figure 4:**
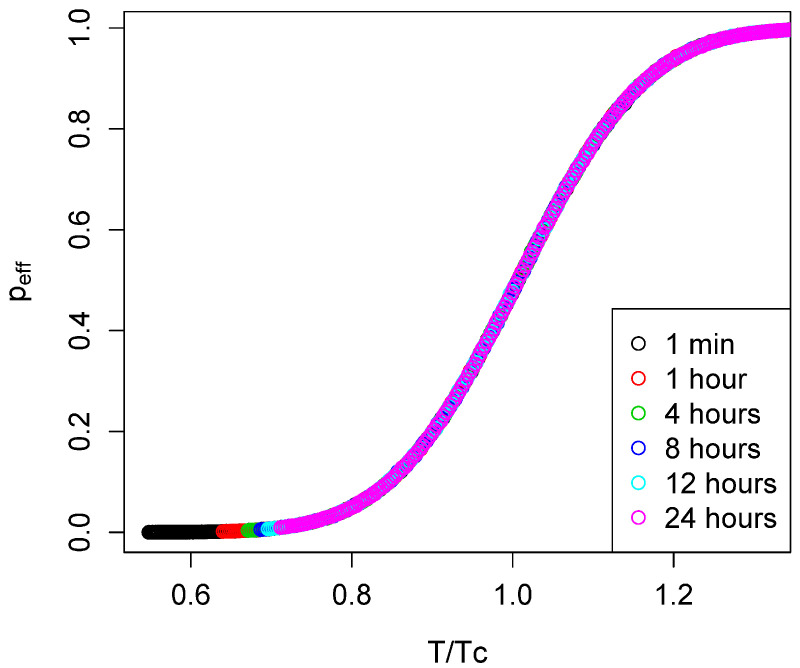
$p_{eff}$ as a function of $T/T_{c}$ for various values of the parameter $t_{exp}$.

The temperature dependence is illustrated in Figure 2. Equation (14) also shows that the temperature dependences of $p_{eff}$ for different $T_{c}$ but the same σ collapse into a common curve when plotted as a function of $T/T_{c}$. This is illustrated by some numerical data in Figure 4.

We suggest that the quite dramatic change in behavior with temperature at $T_{c}$ could have significant implications for evaluating the hypothesis that quenching might have played a significant role in the natural search for lifelike systems on early Earth, as discussed later in the paper.

Another key temperature, here termed $T_{c,2}$, describing the equilibrium distributions was defined and discussed in [7]. At the Gibbs limit, in which the term −1 in the denominator of Equation (2) can be ignored, systems in equilibrium at temperature $T=T_{c,2}$ have a flat $\bar{N_{L}}$ equilibrium distribution; for $T>T_{c,2}$, $d\bar{N_{L}}/dL>0$, and at equilibrium with $T<T_{c,2}$, $d\bar{N_{L}}/dL<0$. $T_{c,2}$ is expressed in terms of the model parameters as

(15) $$T_{c,2}=-\Delta /k_{B}\ln b.$$

In [7], we noted that our earlier analysis [29] of the proteomes of known prokaryotes had shown that proteins in these 4555 prokaryotes had length distributions very close (very small $R_{T}$) to an equilibrium distribution at $T=T_{c,2}$. Note that $T_{c}$, as defined here, characterizes the kinetic behavior of the model, whereas $T_{c,2}$ characterizes its equilibrium properties.

The value that we use for −Δ in (15) in application in laboratory and oceanographic data analyses in the next sections was extracted from [22], which reports data on the equilibrium bond strength of glycine–glycine bonds. To optimize the conditions, leading to quenches which produce large numbers of long polymers, we will require, in these applications, that the temperature of the fluid before quench be larger than $T_{c}$, so that the system will have $p_{eff}\approx 1$ (for rapid ‘sampling’ rearrangements), and also larger than $T_{c,2}$, so that the low-temperature system after quench contains many long polymers. This is further discussed in Section 4 and Section 5, where we compare model predictions with laboratory experiments and oceanographic observations.

To take approximate account of the pH dependence, we use the results in reference [33], where experimental results for the rate of scission of the glycine–glycine peptide bond by hydrolysis are reported for one temperature as a function of pH. The modification of the rate as a function of pH can be described as $\left(k_{pH}/k_{neutral}\right)\times \left(v\;at\;neutral\;pH\right)$, where the values of $k_{pH}/k_{neutral}$ from [33] are shown in Table 1. Thus, the lower limit of the integral on dP/dv, which determines $p_{eff}$ in Appendix C, is modified to $v=\left(k_{neutral}/k_{pH}\right)/\left(f_{a}t_{exp}\right)$. The physical effect of this is that fewer reactions are left out (larger $p_{eff}$) because the rates at highly basic and highly acidic pHs are higher than those at a neutral pH.

With $p_{eff}$ thus fixed, we then proceed, much as in our previous models, to form networks and simulate them dynamically. We use $p_{eff}$, as *p* was used in previous models to decide during network formation whether to include a reaction as described in Appendix A. Each reaction in the network is randomly assigned one enzyme from the species present in the network as in [7]. The complete network formation algorithm, which is different in some details from those used in our previously reported work, is described in Appendix A.

During the dynamical simulation of each network, as described after Equation (9), the simulated systems are ‘fed’ by maintaining the population of dimers and monomers above a specified minimum. (Thus, the system is ‘open’ [34].) The system is continually driven towards equilibrium with the external thermal bath, but many simulated systems do not achieve either local equilibrium or equilibrium with the external bath because of the kinetic blocking imposed by $p_{eff}<1$ and because of the ‘feeding’. As in our previous work, including that described in [7,25,26], we assume that lifelike chemical systems will be metastable states far from equilibrium and select and count such states to obtain a quantitative indication of how likely our models are to result in lifelike states.

As in [26,29], we compute two Euclidean distances $R_{L}$ and $R_{T}$ in the $l_{max}$-dimensional space of sets $\left\{N_{L}\right\}$, which characterize how far the system of interest is from the two kinds of equilibria described above:

(16) $$R_{L}=\sqrt{\sum_{L}{\left(N_{L}-<N_{L}\left(\beta \left(E,N\right),\mu \left(E,N\right)\right)>\right)}^{2}}/\left(\sqrt{2}N\right)$$

for the distance from the locally equilibrated state, and

(17) $$R_{T}=\sqrt{\sum_{L}{\left(N_{L}-<N_{L}\left(\beta ,\mu \left(\beta ,N\right)>\right)\right)}^{2}}/\left(\sqrt{2}N\right)$$

for the distance from the thermally equilibrated state. Alternative measures of the degree of disequilibrium in the context of the study of polypeptide systems have been proposed [35] and we have used alternative formulations in references [25,27]. This formulation has the advantage of discriminating between local equilibrium, which would be achieved by the system in isolation, and the global or thermal equilibrium with an external thermal environment, which would be eventually achieved if the system were in contact with an external, equilibrated ’bath’. The latter distinction has provided valuable insights into the nature of the nonequilibrium states found in our quench simulations. A similar Euclidean measure of disequilibrium in the context of prebiotic evolution was also suggested in reference [36]. More details of the simulation methods are described in [26].

As in [7], the simulations for which the results are reported here implement sudden ‘quenches’ of the simulated networks from high to much lower temperatures of an external thermal bath by an abrupt change in the parameters βΔ during the simulations. In the present work, we also need to take account of the change in $p_{eff}$ and this occurs in principle through a change in the parameter $\beta \bar{\Delta_{a}}$.

In the report of the results which follows, we change the values of $\beta \bar{\Delta_{a}}$ and −βΔ from small values to a large ones by increasing β. The choice of small to large values will correspond, in the case that Δ and $\bar{\Delta_{a}}$ do not change, to a quench from a high to a low temperature. We thus refer in the discussion to quenches from a high to a low temperature, but note that for the relevant parameters βΔ and $\beta \bar{\Delta_{a}}$, a similar change might be induced by a rapid change in pH [33].

## 3. Effects of Barriers on Model Results

In this section, we report results of the use of the model focusing on a comparison of the effects of the added features associated with taking activation barriers into account. We compare the model results for the computational model without barriers described in [7] to the computational models with barriers described in the previous section. We use the variables *N* and *V* and Equations (2) through (4) to determine equilibrium states. In the following sections, comparing the results of the model with barriers with experiment and observations, we fix the variable ρ and use (6) and (8). Models with barriers in the two sections are physically identical except for the factor taking account of polymer dilution in $G_{L}$, as discussed in the previous section.

**Figure 5:**
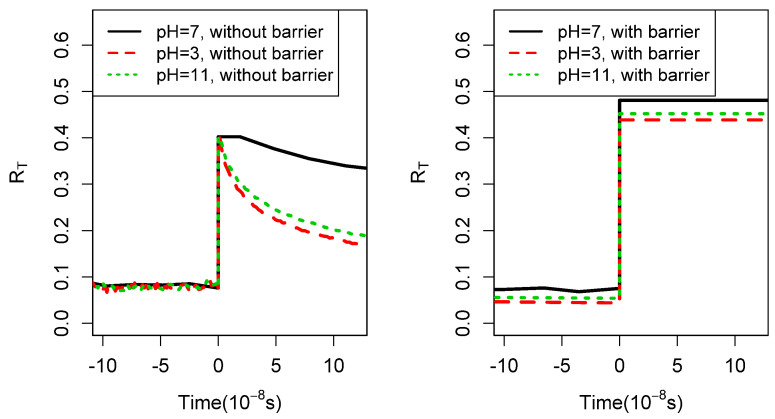
**Left** panel: $R_{T}$ vs. time for short times after a quench in the model of [7]. **Right** panel $R_{T}$ versus time after a quench in the model described in this paper with other parameters unchanged. Upper temperature = 600 K, lower temperature = 280 K, $l_{max}=7,b=4$, $f_{a}=5.96*10^{6}s^{-1}$, $\bar{\Delta_{a}}$ = 108 kJ/mol $\sigma =0.12\bar{\Delta_{a}}$, $t_{exp}$ = 1 year. With these parameters, $T_{c}$ = 350 K and $T_{c,2}$ = 800 K. Each line shows the result of one realization of the same network. $p_{eff}$ is 1 when the temperature is high before quenching.

**Figure 6:**
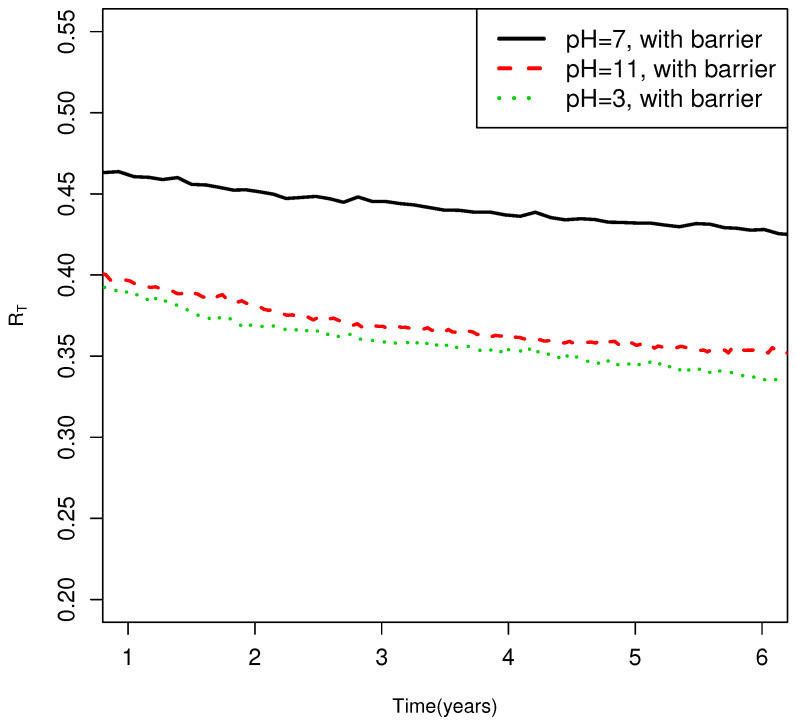
$R_{T}$ as a function of time in the present model after much longer times on the order of years. The same network and parameters as those used in the calculation give the results in the right panel in Figure 5: upper temperature = 600 K, lower temperature = 280 K, $l_{max}=7,b=4$, $f_{a}=5.96*10^{6}s^{-1}$, $\bar{\Delta_{a}}=108$ kJ/mol $\sigma =0.12\bar{\Delta_{a}}$, $t_{exp}=1$ yr. With these parameters, $T_{c}=350\;$ K, $T_{c,2}=800$ K. Each line gives the result of one realization of the same network. peff before quenching is almost exactly 1 with these parameters.

**Figure 7:**
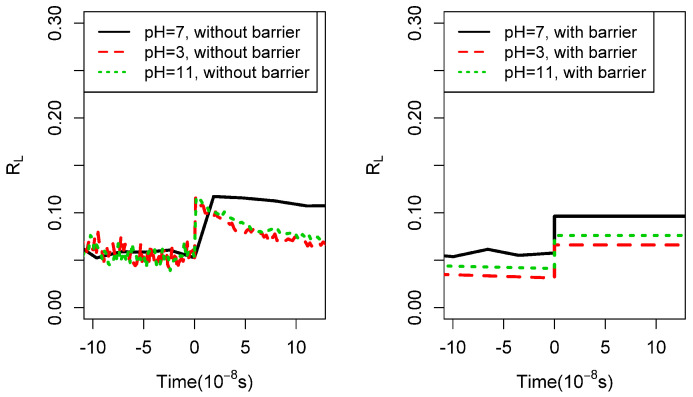
The parameter $R_{L}$ measured in the same simulation leading to the data of Figure 5. Upper temperature = 600 K, lower temperature = 280 K, *l_max_* = 7, *b* = 4, *f_a_* = 5.95 * 10^6>^ s^-1^ , $\bar{\Delta_{a}}$ = 108 kJ/mol, σ = 12 % of barrier, $t_{exp}$ = 1 year, after about 400,000 simulation steps. Each line shows the result of one realization of the same network. The $p_{eff}$ of before the quench is nearly 1. With these parameters, $T_{c}$ = 350 K, $T_{c,2}$ = 800 K.

In Figure 5, we display the results of simulations of the disequilibrium measure $R_{T}$ as a function of real (Gillespie) [31] time for the model without barriers and for the model with barriers for three values of pH. (Other parameters are given in the caption.) One sees a dramatic difference in the time dependence of $R_{T}$ after quenching: on the short time scales displayed, $R_{T}$ is not decaying at all in the new model, whereas it is decaying quite rapidly in a pH-dependent manner when barriers are not taken into account. The disequilibrium (more lifelike) state has been stabilized by the presence of barriers. This has been anticipated by others [37]. On longer time scales, up to years, $R_{T}$ does decay in the new model, but the time scales are much longer, as illustrated in Figure 6. On the other hand, as expected, the system after quenching retains a polymer length distribution close to the one it attained when it was hot, as manifested by a very small change in the parameter $R_{L}$ as shown in Figure 7.

**Figure 8:**
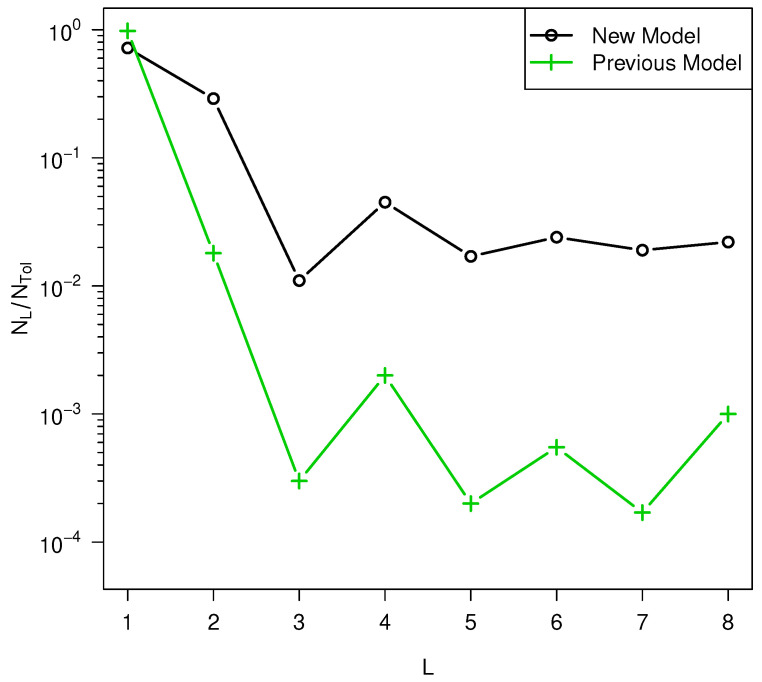
$N_{L}$ versus *L* after quenching for simulations using the model reported here compared with results from the model in [7]. Upper temperature is 373 K, lower temperature = 300 K, *l_max_* = 8, *b* = 2, *f_a_* = 5.95 * 10^6^ s^-1^ , $\bar{\Delta_{a}}$ =108 kJ/mol, σ = 12% of barrier, $t_{exp}$ is 1h. Simulations of about 400,000 reaction steps per run. Results are an average of 100,000 realizations. With these parameters, $T_{c}$ = 430 K, $T_{c,2}$ = 1600 K.

**Figure 9:**
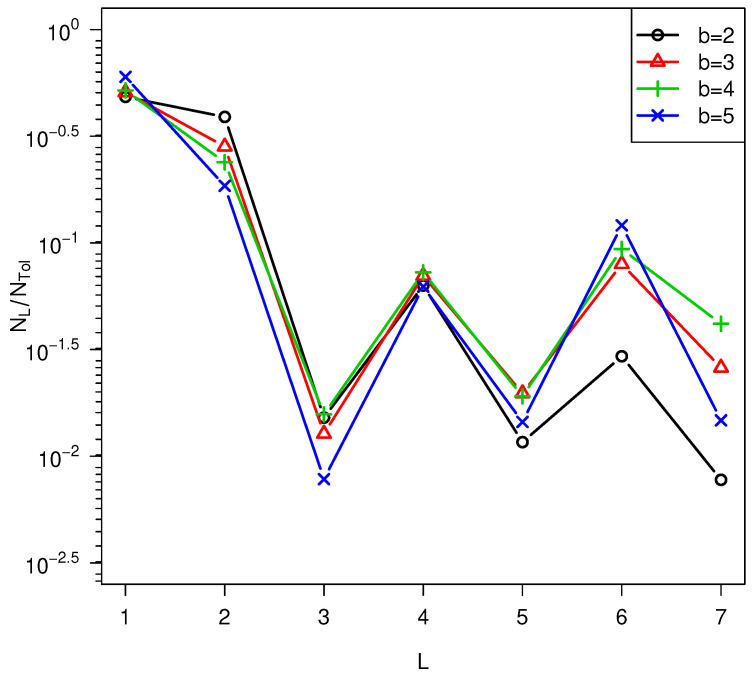
Effect of *b* on $N_{L}$ is small. Simulation parameters $p_{eff}=0.1$, high temperature = 373 K, low temperature = 300 K, $l_{max}$=7, *f_a_* = 5.95 * 10^6^ s^-1^ , $\bar{\Delta_{a}}$ = 108 kJ/mol, σ = 12% of $\bar{\Delta_{a}}$.

**Figure 10:**
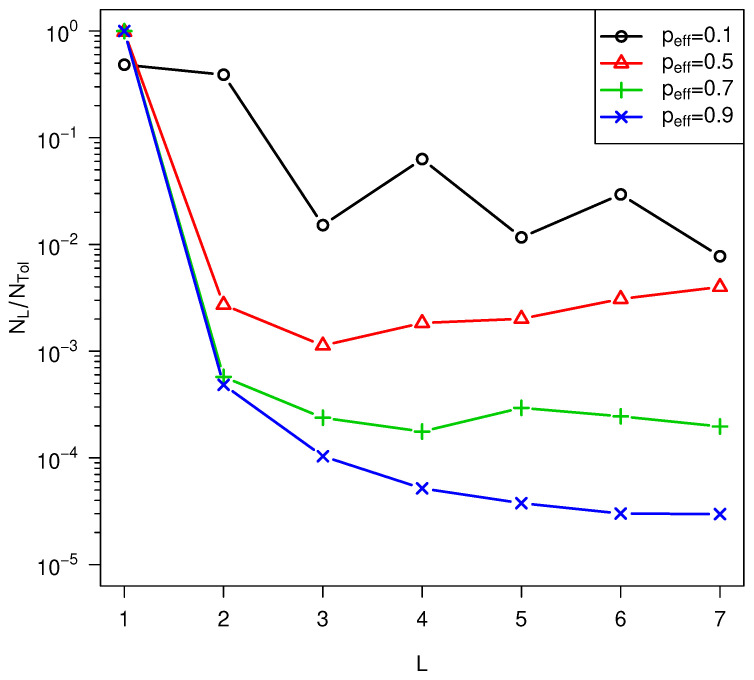
Dependence of the $N_{L}$ distribution on $p_{eff}$b=2, high temperature = 500 K, low temperature = 300 K, $l_{max}$ = 7, *f_a_* = 5.95 * 10^6^ s^-1^ , $\bar{\Delta_{a}}$ = 108 kJ/mol, $\sigma =0.12\bar{\Delta_{a}}$.

The effects on the polymer length distributions of adding barriers to the model are illustrated in Figure 8. The enhancement in the number of long polymers is significant. In this simulation, the parameter b=2 was used. However, as shown in Figure 9, the oscillatory behavior in *L* persists for larger *b* values. On the other hand, increasing $p_{eff}$ by increasing $\bar{\Delta_{a}}$ or reducing $t_{exp}$ causes the oscillatory behavior to disappear, as shown in Figure 10.

## 4. Comparisons with Experiments on Amino Acids Forming Polypeptides

As in [29] and described in Section 2, for comparison with laboratory experiments as described in this section and with oceanographic observations as described in the next section, we need to use the formulation of the model which takes approximate account of the dilution of polymers in solution so that the equilibria are described by Equations (6)–(8) and the parametric inputs are ρ and *e* instead of *N* and *E*.

To compare the simulation model with the experimental data reported in [8,9,10,11], we set the before-quench high temperature and the after-quench low temperature to the values reported in these references. $p_{eff}$ was then adjusted so that the simulations gave the best fit to the data. With $p_{eff}$ thus determined, we used (14) to determine $T_{c}$ with the fixed value of ξ=0.25. Finally, using $f_{a}=5.96*10^{6}s^{-1}$ extracted from [23] and Equation (13), we estimated $t_{exp}$ with $\bar{\Delta_{a}}=108\;\mathrm{kJ}/\mathrm{mol}$. In the last step, the estimated $t_{exp}$ was exponentially dependent on $\bar{\Delta_{a}}$, and the value of $\bar{\Delta_{a}}$ used was adjusted by about 10% relative to the value reported in [23] in order to bring the estimates of $t_{exp}$ into order-of-magnitude agreement with the experimental reports. The main uncertainties in this procedure arise from our disregard of an appropriate value of σ (entering in ξ) and the value of $\bar{\Delta_{a}}$. We found only one carefully reported value [23] for $\bar{\Delta_{a}}$ which was for glycine–glycine hydrolysis. The experiments reported in [8] included alanine as well as glycine in the solution and that could contribute to an uncertainty in $\bar{\Delta_{a}}$ as well as in σ.

**Figure 11:**
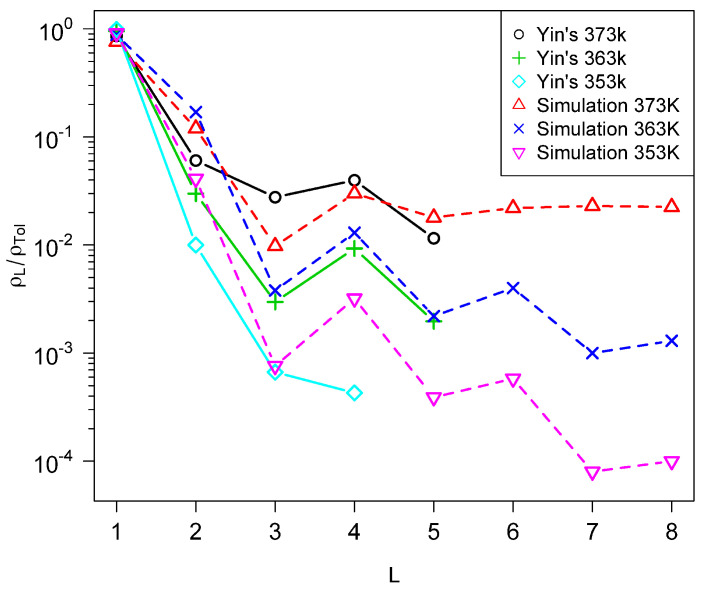
Simulations compared with experimental data from reference [8]. High temperatures are as labeled, low temperature = 300 K, *l_max_* = 8, *b* = 2, *f_a_* = 5.95 * 10^6^ s^-1^ , $\bar{\Delta_{a}}=108$ kJ/mol, $\sigma =0.12\Delta_{a},t_{exp}$ is about 1 h. About 40,000 simulation steps per run. Results are averages of 100,000 realizations. With these parameters, $T_{c}$ is about 490 K and $T_{c,2}$ = 1600 K.

**Figure 12:**
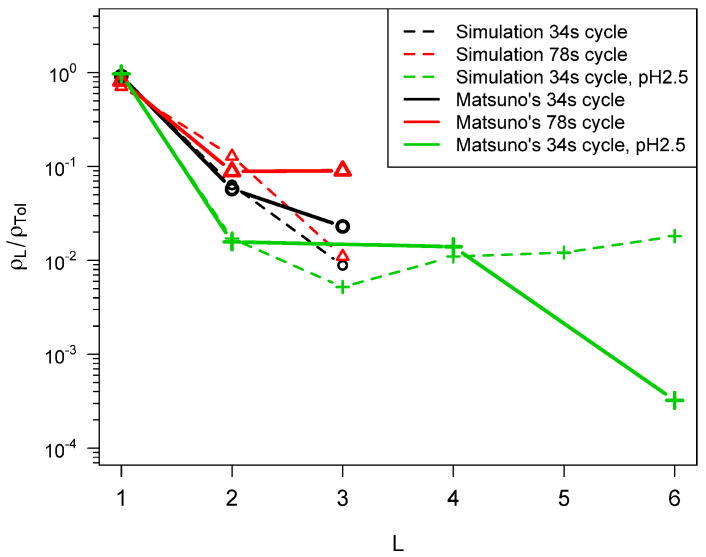
Fit of the simulation model to data from the experiments reported in references [9,10,11]. In the simulations, the high temperatures = 500 K, low temperature = 300 K, *f_a_* = 5.95 * 10^6^ s^-1^ , $\bar{\Delta_{a}}$ = 108 kJ/mol, $\sigma =0.12\bar{\Delta_{a}}$ The fit yields $t_{exp}=12$ s for Matsuno’s 34 s cycle and $t_{exp}$ 9 s for the 78 s cycle. Simulation results are averages of 10,000 realizations. With these parameters, $T_{c}$ is about 590 K and $T_{c,2}$ = 1600 K.

Figure 11 shows fits to the experiments described in [8], and Figure 12 shows fits to the data reported in [9,10,11]. The same values of $\bar{\Delta_{a}}$, ξ, and $f_{a}$ were used in all the calculations. The oscillations in $N_{L}$ as a function of *L* seen in [8] are quite well reproduced by the model and the fits are quite good at the higher initial temperatures. Notably, the orders of magnitude of the ratios are quite well reproduced, whereas they were as much as two orders of magnitude lower using the previous model without barriers.

**Figure 13:**
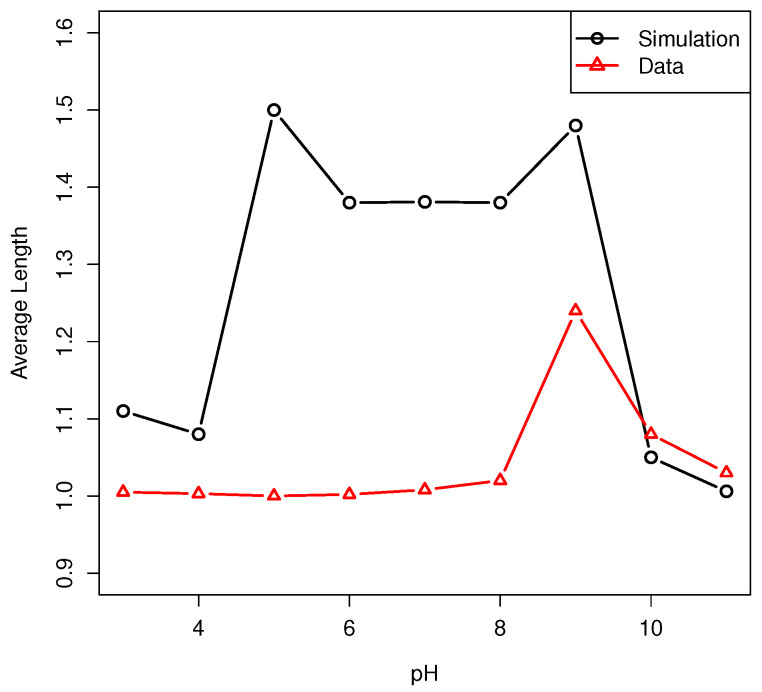
pH dependence of the average polymer length produced in the simulations and in the experiments of [8]. $p_{eff}$ = 1, high temperature = 373 K, low temperature = 300 K, $l_{max}=8$, b=2, *f_a_* = 5.95 * 10^6^ s^-1^ , $\bar{\Delta_{a}}=$ 108 kJ/mol, $\sigma =0.12\bar{\Delta_{a}}$.

We performed an extensive comparison of the pH dependence of the predicted polypeptide length distribution with that reported in [8]. To summarize, the predicted enhancement of the amount of polypeptides produced in alkaline solution is in reasonable agreement with that reported in [8], but the model predicts a much higher enhancement in polypeptide formation in acid media than reported in [8]. In Figure 13, we show the average polypeptide length as reported in [8] and the corresponding result of the simulations to illustrate this result. The model pH dependence is parametrized by the experimental results in [33]. The solution chemistry appears to be nearly the same in the two sets of experiments reported in [8,33], and we were unable to determine the source of this discrepancy. It is discussed somewhat further in Section 6.

## 5. Application to Quenches in Ocean Rifts

We have previously reported evidence [29] from values of $R_{T}$ and $R_{L}$ for proteomes of 4555 prokaryotes that the proteins in these organisms were formed at temperatures on the order of 370 K. Most of the prokaryotes in this sample are not thermophilic, so our analysis suggested that the proteins, and not necessarily the full prokaryotic organisms, were formed at that high temperature. There are at least two possibilities concerning the possible order of events here. The proteins could have formed in a quench from amino acids in the waters emitted from an ocean ridge or hot spring and then, after the quench and in rare cases, formed prebiotic entities with some of the properties of prions or amyloids in the contemporary biosphere. Alternatively, one might consider models in which the proteins were formed at high temperature and then, before the quench, became part of a thermophilic prokaryote. In either scenario, the formation of suitable polymers (proteins, RNA, or others) at high temperature is the first, probably rate-limiting, step in prebiotic evolution. In the model considered here, we consider only this step and hypothesize that the formation of precursor polymer assemblies which could evolve into life only occurs after the quench. We do not attempt to model later processes in detail here.

Motivated by the perspective described in the last paragraph, we therefore report a few results here using the model described in the last section with parameters suggested by oceanographic studies of smokers in or near ocean rifts. Although the stability of amino acids in the hot fluids in hydrothermal environments has been questioned, laboratory measurements [38], as well as free energy calculations reported by Shock and coworkers [37], suggest that amino acids could be stabilized in such fluids in the presence of hydrogen and they are in fact observed to be present.

The concentrations of amino acids in the fluids emitted from smokers have been measured [24] in six black or white smokers in the Mariana Trough, and were reported to be up to more than $10^{-5}$ molar of total amino acids and $10^{-8}$ molar or less of dissolved free amino acids. This implies that most of the amino acids could be inferred to be in polypeptides. The large number of detected amino acids in large molecules (presumably polypeptides) could suggest a biological origin, but the authors of [24] observed that higher-temperature smokers exhibit a higher concentration of long polymers. The temperatures in these high temperature smokers exceed the maximum temperature at which thermophilic bacteria can survive, so, in these high-temperature smokers, the polypeptides observed probably have an abiogenic origin.

Note that in the scenario explored in the model considered here, long polymers, of which some may turn out to be capable of supporting prebiotic evolution, only form transiently in the high-temperature stage of the hypothesized quenches. The role of the quench in this model is to stabilize the long polymers which were transiently present in the hot stage, and selective evolution, if it occurs, occurs in the low-temperature stage. The advantage, in our view, of this scenario is that it permits both a rapid sorting through many randomly selected types (for example, polypeptides) in the hot stage, while the quenches continuously ’sample’ them into a cooler environment which may permit them to evolve. Thus, in this model, we do not require that any lifelike entities survive in the hot stage before the quench. The model predicts that high temperatures in the hot stage can enhance long polymer formation (because entropic effects are dominant), and we therefore expect that hot stages in which the temperatures do not permit any known hydrophilic organisms to survive may nevertheless be favorable for the production of prebiotic material, leading to lifelike development after the quench.

Temperatures of what we interpret as the fluid temperature before the quench taking place in the smokers were reported in [24] to be up to 530 K. pH values were acidic, in the range of 3.1 to 5.5. As in the laboratory experiments, the least well-known parameters are the width of the Gaussian distribution of barriers to hydrolysis and the dwell time of the fluids at high temperature before the quench. Glycine was the most common amino acid in the oceanographic samples, followed by serine, asperine, and lysine. This might suggest that the values of the width σ which were used to fit the laboratory data and the values of $\bar{\Delta_{a}}$ and $f_{a}$ as reported in [23] in glycine–glycine hydrolysis could be used, and we have applies them here. The dwell times of the fluids at high temperature before the quench are unknown for smokers, but models [39] suggest much longer times (on the order of up to $10^{5}$ years) than those experienced in the laboratory experiments. As noted earlier, larger values of $t_{exp}$ lower the value of $T_{c}$, so it is more likely that the temperature before quenching will exceed $T_{c}$ if the other parameters are the same.

**Figure 14:**
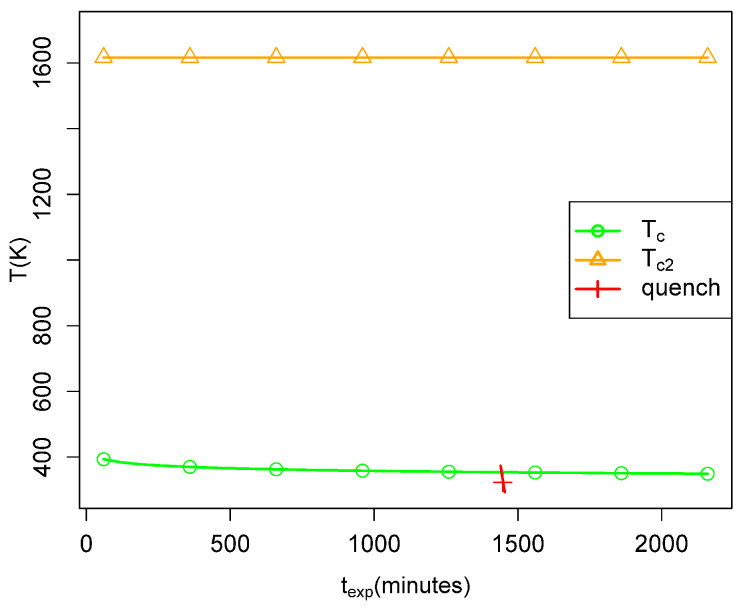
$T_{c}$ versus $t_{exp}$ for parameters typical of the discussed laboratory experiments. The diagonal brown line indicates approximate the bath temperature during a quench. The yellow horizontal line is $T_{c,2}$ when b=2.

**Figure 15:**
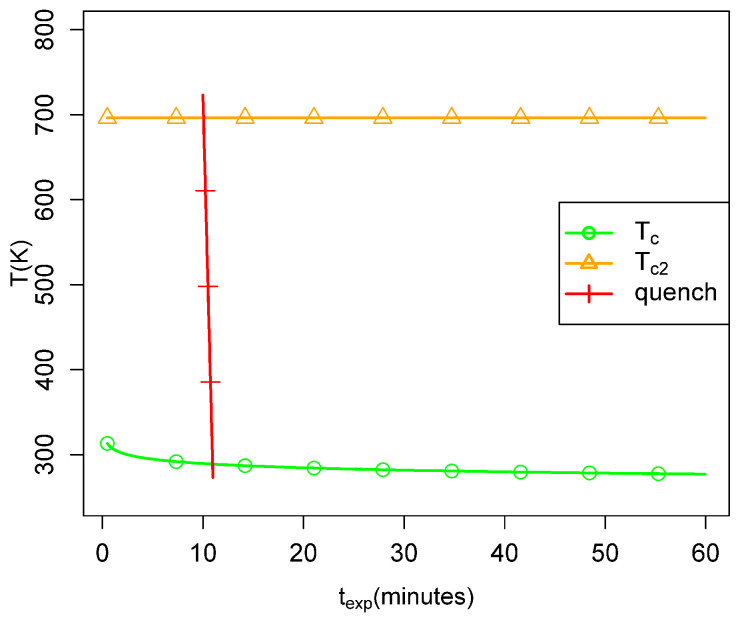
Same as the previous figure but with parameters expected to be characteristic of the quenches occurring in the smokers in the Mariana Trough. $T_{c}$ is likely to be crossed during the quenches as suggested by the red line. The yellow horizontal line is $T_{c,2}$ when b=5.

Quantitatively, this point is illustrated in Figure 14, which plots the values of $T_{c}$ and $T_{c,2}$ for a range of dwell times expected in the laboratory experiments with fixed values of $\bar{\Delta_{a}}$ and $f_{a}$. In Figure 15, we show the corresponding relationship using parameters approximating the conditions in the smokers. One sees that the laboratory experiments are not likely to have taken the fluid from above to below $T_{c}$ (that is, from $p_{eff}\approx 1$ to $p_{eff}\approx 0$), whereas the quenches in the smokers are very likely to do so. However, for experiments or observations to yield long polymers after quenching in the smokers, we also need a temperature before quenching which is above $T_{c,2}$, so that many long polymers are present in the fluid before quenching. One sees in Figure 15 that over much of the ranges of *b* and $t_{exp}$ of interest for the smokers, the second requirement is more difficult to satisfy, but it may be satisfied in the highest-temperature smokers.

What was actually measured in the observations of [24] was the total number of amino acids and the number of amino acid monomers. If the temperature before quenching is above $T_{c}$, then $p_{eff}$ is close to 1 and the quench to low temperatures will lead to a length distribution characteristic of equilibrium at the temperature before quenching. These conditions appear to be met in all the smokers for which data were reported in [24]. If the factor $L^{-3\nu }$ in Equation (6) is ignored (or equivalently, ν=0), then the the predicted ratio $\sum_{L=1}^{l_{max}}N_{L}/N_{1}$ can be evaluated analytically at the Gibbs limit, as shown in Appendix D. If the hot temperature is below $T_{c,2}$, one can take the limit $l_{max}\to \infty$, and the sum in the numerator converges. However, for temperatures above $T_{c,2}$, the sum diverges in that limit and an infinite value of the ratio would be predicted if no further physics constrained the values of *L* to a finite maximum. These features are retained when the sum including the factor $L^{-3\nu }$ is retained and the sum is evaluated numerically. However, for *b* values between 5 and 10, we find that the temperatures before quenching are somewhat below $T_{c,2}$.

**Figure 16:**
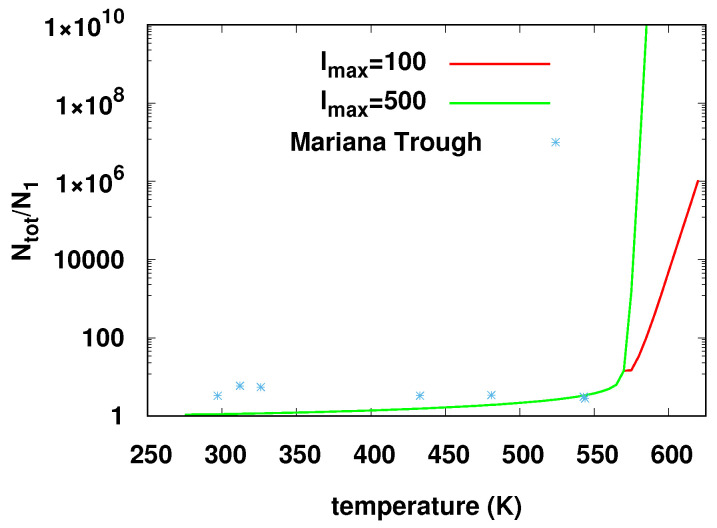
The ratio of the total number of amino acid molecules to the number of monomeric amino acids for two values of the maximum polymer length ($l_{max}$) in the model compared with values from observational oceanographic data from reference [24]. We took b=7 and pH=7 here. The corresponding value of $T_{c,2}$ is 570.4 K. Above $T_{c,2}$, the model value of the ratio diverges as $l_{max}\to \infty$. In the figure, one sees a slight decline in the ratio just above $T_{c,2}$ in the case of $l_{max}=100$, which arises from the factor $L^{-3\nu }$ in Equation (6).

We compared the calculated ratio with the reported observations with various values of $l_{max}$ and *b*. In Figure 16, we show the results for two values of $l_{max}$ and b=7, for which $T_{c,2}$= 570.4 K. It is not completely clear what value of *b* should be used for this comparison. The tables in [24] list eleven amino acids, but some of them are present in much smaller quantities than others. A model assigning different probabilities for different monomer types is possible, but we have not studied it here.

We can draw these limited conclusions from this comparison: Most of the observational data are associated with temperatures below the most likely values of $T_{c,2}$, and at these temperatures, the model predicts ratios larger than 1 but smaller than those observed. A few of the observational data points are associated with temperatures which may be greater than the range of expected values of $T_{c,2}$. At these temperatures, the model with $l_{max}\to \infty$ predicts an infinite ratio and by arbitrary adjustment of $l_{max}$, one could obtain a theoretical result quite close to the observations. However, a physical theory is needed which takes into account physical factors which will limit $l_{max}$ to finite values.

The authors of [24] point out that at the highest temperature values seen, thermophilic organisms which can survive are not known. Thus, a possible understanding of these data could attribute the relatively large values observed at lower temperatures to biogenic origins of the observed polypeptides, which our model does not take into account, whereas at the highest temperatures, the ratio must be fixed by abiogenic ligation, which the model does take into approximate account. (As noted above, high-temperature quench stages at which thermophilic organisms cannot survive are not excluded from relevance to prebiotic evolution within the model considered here. We regard the hot stage as producing long polymers which survive transiently at high temperatures but which are stabilized by the quench at lower temperatures where prebiotic evolutionary processes would have time to take place.) With regard to the data shown in Figure 16, we can attain at a possible qualitative understanding of the fact that the model agrees better with the (limited) observational data at high temperatures where no biogenic polymers are expected. In summary, we find that the model appears to agree semiquantitatively with the very large difference (about two orders of magnitude) between the ratios observed in the laboratory experiments and those observed in the oceanographic data.

## 6. Discussion and Conclusions

We present an extension of an earlier model [7] for prebiotic formation of biomolecules on early Earth, in which the molecules formed by ligation from starting solutions of monomers at high temperatures were then quenched rapidly at lower temperatures so that they retained the polymer length distribution attained at the high temperature before the quench. The model might apply to either quenching from hot fresh water springs or from emissions from ocean floors. In either case, it may suggest a way to evade the issue sometimes raised concerning thermophilic origin of life scenarios, namely that the needed long polymers would not survive long enough at high temperatures to permit evolution to initiate. At the high temperature before quenching in our model, there are many long polymers under the conditions discussed, but they are rapidly disintegrating and reforming by scission and ligation, making evolutionary development unlikely if the temperature remains high. In the quench to cooler temperatures however, the population of long polymers is retained, while the rapid scission and ligation stops, thus allowing time for evolutionary selection, possibly leading to lifelike states. These general ideas were suggested in our earlier paper [7]. Evidence for a high-temperature origin of prokaryotic proteomes was reported in our earlier study [29] of experimental data on modern prokaryote proteomes. It is consistent with genomic evidence cited much earlier by others [40] for some sort of thermophilic origin of life. In the scenario suggested here, the high-temperature period in the origin of life occurs in the formation of the basic biomolecules required, before any of the elaborate apparatus of modern cells emerged.

In the present paper, we have extended the model of [7] to take account of the known fact that hydrolysis and ligation rates of biopolymers are limited by free energy transition barriers. Barrier heights are selected from a Gaussian distribution centered near the measured barrier height for glycine–glycine hydrolysis. The finite width of the barrier distribution produces the network sparseness, which is partially responsible for the metastability of the quenched states predicted. The new model greatly extended the predicted stability of the quenched states, as illustrated in Section 3. We also introduced a phenomenological parameter which permits the effects of pH on the results to be incorporated. The needed parametrization is taken from [33]. The pH dependence of the results is approximately symmetric about pH 7.

We report a comparison of the results of the model with two sets of experiments [8,9,10,11] and with some oceanographic data on polypeptide populations from oceanic smokers in the Mariana Trough [24]. The data from [8] yield a polymer length distribution which fits quite well with the predictions of the model at neutral pH and, in particular, gives a fitted value of the time $t_{exp}$ spent at high temperature before the quench which is consistent with that reported in the experiments. Also, notably, the number of polypeptides reported to be produced is small compared to the number of amino acid monomers (glycine and alanine) remaining after the quench, consistent with the model predictions for the high and low temperatures reported for the experiments. The model also agrees with the enhancement in the number of polypeptides produced at alkaline pH observed in [8]. However, our model predicts a significantly larger enhancement in polypeptide production at acidic pH than is reported in [8]. Our parametrization of the pH dependence depends on the data in [33], but we have not been able to trace the chemical differences between the systems used in the experiments of [8] and those of [33] that might account for the difference. Possibly the fact that the experiments reported in [8] included both alanine and glycine, whereas those in [33] only addressed glycine–glycine hydrolysis is relevant. Strictly speaking, the experiments in [8] are not quenches in exact conformity with what is modeled here. They are ’drying’ experiments, in which the solution is held at a high temperature and then dried. This drying will result in cooling and in stopping the reactions, but the physical situations after the quench are not the same as in our model, nor are they the same as the physical situation after quench in the emissions from an ocean trench. However, this does not suggest to us any clear explanation for the discrepancy in the results with the model ones under acidic conditions.

Comparing with the more limited data from the Mariana Trough [24], a striking observation is that, in sharp contrast to the laboratory experiments, [24] reports many fewer monomeric amino acids relative to the total number of amino acids in their samples. Our model is qualitatively, and even semiquantitatively, consistent with that result, attributed to the higher temperatures and longer ‘dwell times’ ($t_{exp}$) experienced by the fluids before they emerge from the smokers in the Mariana Trough.

The model makes several falsifiable predictions. In particular, it predicts the dependency of polymer length distributions on the dwell time of the fluid at high temperature before quenching, the temperature before quenching, and the number of types of available monomers (amino acids for polypeptides) that can be compared with future oceanographic observations and laboratory experiments.

## Figures and Tables

**Table 1:** $k_{ph}=k_{ph=n}/k_{ph=7}$.

pH	3	4	5	6	7	8	9	10	11
$k_{ph}$	17.78	2.78	1.18	1	1	1	1.12	2.22	12.22

**Figure A1:**
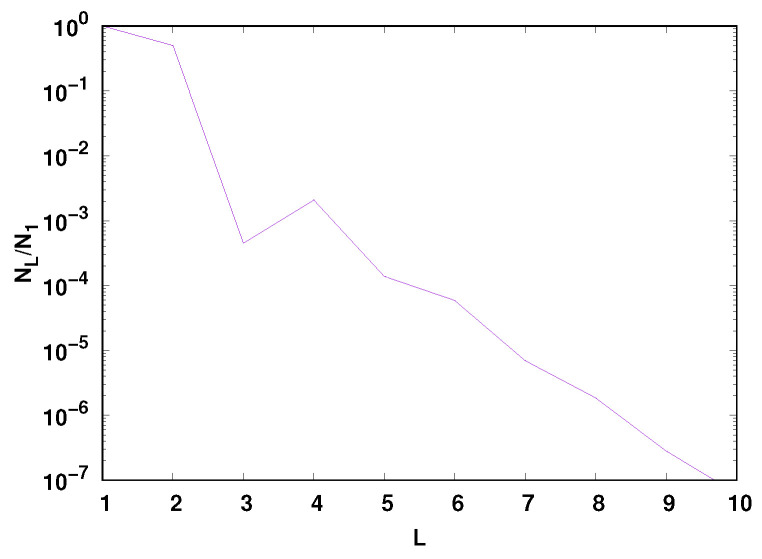
$N_{L}/N_{1}$ in the mean field model with a parametrization giving oscillations qualitatively similar to those observed. b=1,c=0.1,d=0.05. Initial conditions $N_{2}/N_{1}=0.5$. Here, we summed the two small real solutions $u_{1}$ and $u_{3}$ of the cubic equation in the form $u_{1}^{L}+3u_{3}^{L}$ with L=3,…,10. With the parameters cited, the roots $u_{1}$ and $u_{3}$ were $u_{1}=0.1892$ and u=−0.1282.

## Data Availability

Software and data associated with this work is publicly available from the Minnesota Conservancy at https://conservancy.umn.edu/handle/11299/228099.

## Appendix A. Algorithm for Generation of the Networks

This is nearly the same as the algorithm described in reference [25] but differs in the following details: the *p* value is selected differently and the list of reactions from which reactions are chosen does not include enzymes, which are instead assigned during the network formation process.

Algorithm:

- Determine the parameter $p_{eff}$ using the values of the average activation energy, its mean square deviation, and the time which the system will spend in the current thermal condition as described in Section 2. Select the parameter $l_{max}$, the maximum length of polymers to be considered. $p_{eff}$ is the probability that a possible reaction in the network is actually included in the network (as described in more detail below).
- Select a ‘firing disk’ of initial short polymers to include in the network.
- From all ligation and scission reactions which can occur involving polymers already in the network and other polymers of length less than or equal to $l_{max}$ and which have not yet been marked, select one at random. This is implemented as follows.
- Select a polymer already in the network at random.
- Choose ligation or scission with equal probability.
- If scission, select a connection at random along the string (among L−1 ‘bonds’ if the string has length *L*) for scission.
- If ligation, choose another polymer at random from among all possible polymers for connection.
- In either case, select an enzyme at random from polymers already in the network.
- Check to determine if the reaction selected has already been tried. If so, check to determine if the number of reactions already tried is equal to the total number of available reactions. If so, stop. Do not include the network but count it as an attempt to create a network. If not, include the reaction in the network with probability $p_{eff}$. Whether it is included or not, mark that reaction as ’tried’ and go back and pick another reaction.

## Appendix B. Mean Field Model for Polymer Length Distribution

Here, we demonstrate that the damped even–odd oscillations observed in experiments of Yin et al. [8] arise in a simple model for the reaction kinetics as long as the population of monomers and dimers is much larger than the population of longer polymers and all the reactions in which a dimer or a monomer is added or removed from a chain of any length *L* occur in the network.

Note that these conditions restrict the relevance of this model to the laboratory experiments considered in this paper. In the oceanographic data we considered, the number of monomers is reported to be much *smaller* than the total number of polymers and the first condition above is probably not met. Also, the inclusion of a large number of dimers as well as monomers here is essential. If only reactions in which monomers are added or removed from any chain are included, then the model described in this appendix does not predict the oscillations we report below. The oscillations in the solution we find are an even–odd effect resulting from competition between the effects of adding or deleting monomers from polymers with the effects of adding or deleting dimers from these polymers. Thus, the period of these oscillations is always one monomer unit. As mentioned briefly at the end of this appendix, the parameter space includes regions in which the model has solutions with oscillations with a continuum of real frequencies, but our estimates of the parameters associated with the reported experiments are not in that parameter region. The experiments appear to have only even–odd oscillations, so with regard to this feature, the model agrees with the experiments.

Under the stated assumptions, the rate at which the population $N_{L}$ of polymers of length *L* changes with time is

(A1) $$\begin{array}{cc} dN_{L}/dt= & \\ K_{\to }^{\left(1\right)}N_{L+1}-K_{\leftarrow }^{\left(1\right)}N_{1}N_{L}+ & K_{\leftarrow }^{\left(1\right)}N_{L-1}N_{1}-K_{\to }^{\left(1\right)}N_{L}\\ +K_{\to }^{\left(2\right)}N_{L+2}-K_{\leftarrow }^{\left(2\right)}N_{2}N_{L}+ & K_{\leftarrow }^{\left(2\right)}N_{L-2}N_{2}-K_{\to }^{\left(2\right)}N_{L} \end{array}$$

Here, the subscript → refers to scission, which is the ‘forward’ reaction for peptide bonds, and ← refers to ligation. Eight x $N_{max}-2$ reactions are kept. We assume that $N_{1}$ and $N_{2}$ are fixed at large values, as they will be in many experiments and observations. We denote $x_{L}=N_{L}/N_{1}$, $b=K_{\to }^{\left(1\right)}/K_{\to }^{\left(2\right)}$, $c=K_{\leftarrow }^{\left(1\right)}N_{1}/K_{\to }^{\left(2\right)}$ $d=K_{\leftarrow }^{\left(2\right)}N_{2}/K_{\to }^{\left(2\right)}$ and seek a steady-state solution of the equations giving

(A2) $$1x_{L+2}+bx_{L+1}-\left(1+b+c+d\right)x_{L}+cx_{L-1}+dx_{L-2}=0$$

for L>2. This is a fourth-order homogeneous difference equation which has solutions of form $x_{L}=u^{L}$ with four solutions for *u*. u satisfies the fourth-order polynomial equation

(A3) $$u^{4}+bu^{3}-\left(1+b+c+d\right)u^{2}+cu+d=0$$

An obvious solution is u=1. Dividing out u−1 gives the third-order polynomial equation

(A4) $$u^{3}+\left(b+1\right)u^{2}-\left(c+d\right)u-d=0$$

The analytical form of the solution is complicated [41], but the essential features are illustrated by plotting the polynomial for small, real, positive values of the parameters *c* and *d*. There are three real roots. Two are negative and one is positive. Two of the roots are nearly symmetrically positioned around zero and the third is larger and negative. The negative roots give even–odd oscillations in the populations as a function of *L*. A linear combination of the two small roots, which gives $N_{L}$ as a function of *L* similar to the results of the experiments and to our numerical simulations of the full model, is shown in Figure A1. Here, we used the known analytical solution to the cubic to determine the roots [41]. When the discriminate $D={\tilde{b}}^{2}/4+{\tilde{a}}^{3}/27$ is negative, the roots are all real; otherwise (D>0), one root is real and two are complex. In the present case, $\tilde{b}=\left(2/27\right){\left(b+1\right)}^{2}+\left(1/3\right)\left(b+1\right)\left(c+d\right)-d)$ and $\tilde{a}=-\left(c+d\right)-{\left(b+1\right)}^{2}/3$. With the parameters used in the figure, D=−0.0853, suggesting that in some physically realizable contexts, one might find the second type of solution with D>0. *b* is expected to be close to 1, but a physical argument restricting *c* and *d* to values giving D<0 is not evident to us.

## Appendix C. Form of the Probability Distribution for *v* and Expression for *p_eff_*

We take all the rate parameters *v* in the master Equation (9) to have the form $v=e^{-\Delta_{a}/k_{B}T}$ with $\Delta_{a}$ confined to the range $0<\Delta_{a}<\infty$. Over this (restricted) range, $\Delta_{a}$ is assumed to be Gaussian distributed about an average value $\bar{\Delta_{a}}$. The constraint to positive $\Delta_{a}$ is required to avoid unphysical negative activation barriers which would lead to v>1. Very small $\Delta_{a}$ values lead to very high (nearly barrierless) reaction rates, seldom realized in numerical practice because they are very improbable, but the constraint must be taken into account in the normalization of the Gaussian. The distribution of $\Delta_{a}$ is

(A5) $$dP/d\Delta_{a}=Nexp\left(-{\left(\Delta_{a}-\bar{\Delta_{a}}\right)}^{2}/2\sigma^{2}\right)$$

where the inverse of the normalization constant N is

(A6) $$N^{-1}=\int_{0}^{\infty }exp\left(-{\left(\Delta_{a}-\bar{\Delta_{a}}\right)}^{2}/2\sigma^{2}\right)d\Delta_{a}$$

differing from the usual normalization in the lower limit. Transforming to the dimensionless variable $u=(\Delta_{a}-\bar{\Delta_{a}}/\sigma$ and using the definition of the error function erf(z), one finds

(A7) $$N=\frac{{\left(2/\pi \right)}^{1/2}}{\sigma (1+erf\left(\bar{\Delta_{a}}\sqrt{2}/\sigma \right))}$$

which reduces to the usual factor $1/({\left(2\pi \right)}^{1/2}\sigma )$ for a Gaussian distribution with the limit $\bar{\Delta_{a}}\to \infty$ as expected. In practice, the argument of the error function here is large but the correction is important in correctly normalizing the definition of $p_{eff}$. With this normalization, we use the relation $v=e^{-\Delta_{a}/k_{B}T}$ and $dP/dv=\left(dP/d\Delta_{a}\right)\left(d\Delta_{a}/dv\right)$ and find the form in Equation (11). Note that the parameter σ is not exactly the variance of this renormalized distribution, nor is $\bar{\Delta_{a}}$ exactly the average value of $\Delta_{a}$ but, in practice, the (computable) corrections to the variance and average are negligible.

The definition of $p_{eff}$ as described in the text is

(A8) $$p_{eff}=\int_{1/(t_{exp}f_{a})}^{1}\left(-dP/dv\right)dv$$

Using Equation (11), transforming back to the variable $\Delta_{a}$ and then to the dimensionless variable $t=\left(\Delta_{a}-\bar{\Delta_{a}}\right)/\sqrt{2}\sigma$, and using the definition and properties of the error function as well as Equation (13) for $T_{c}$, we find:

(A9) $$p_{eff}=\frac{erf\left(\xi \right)-erf(\xi \left(1-T/T_{c}\right)}{1+erf(\xi )}$$

where $\xi =\sqrt{2}\left(\bar{\Delta_{a}}/\sigma \right)$. This expression has the right limits as T→0 and T→∞.

## Appendix D. The Measured Ratio from Smoker Data as Predicted by the Model

As explained in the text, we anticipate that the high temperatures found in the smokers are all well above $T_{c}$. Therefore, $p_{eff}$ will be close to 1 in our model and the observed distribution at low temperatures after quenching to ocean bottom temperatures will be close to the equilibrium distribution at the initial high temperature. To calculate the expected ratio of the total number of amino acids to the number of monomeric amino acids, we use the Gibbs limit of Equation (2), in which the term −1in the denominator is ignored. If we set ν=0, then the sum is geometrical and easily evaluated, giving a ratio of

(A10) $$N_{total}/N_{1}=\left(\frac{e^{-\Delta \beta }}{\left(b-1\right)})\right)\left[\frac{e^{\alpha (l_{max}+1)}-1}{e^{\alpha }-1}-\frac{e^{\Delta \beta (l_{max}+1)}-1}{e^{\Delta \beta }-1}\right]$$

where

(A11) $$\alpha =-\Delta \beta (1-T/T_{c,2})$$

When $T<T_{c,2}$, α<0 and the limit $l_{max}\to \infty$ converges to a finite result for the ratio. When $T>T_{c,2}$, this limit results in an infinite ratio which is unphysical. For ν≠0, the sum is not geometric and has been numerically evaluated to produce the results displayed in Figure 16, where the divergence when $T>T_{c,2}$ is again evident. As noted in the text, in the case of $T>T_{c,2}$, we need an account of additional physics not in the model to account for a limited maximum length. However, with b=7 and using the glycine–glycine bond energy, we find $T_{c,2}=570.4$ K, and the hot smoker temperatures reported in [24] are all below (though in some cases not far below) $T_{c,2}$, so that we can use the calculated result for the ratio without specifying a value for $l_{max}$ for a comparison with the data. The result is plotted as a function of the hot temperature before quenching in Figure 16 using b=7 and $T_{c}=570.4$ K, where it is compared with the oceanographic data from the Mariana Trough.
